# Long-term outcomes of surgical treatment of cervical spine involvement in rheumatoid arthritis

**DOI:** 10.1007/s00264-025-06654-6

**Published:** 2025-09-20

**Authors:** Andrés Combalia, Apol·lònia-Maria Salvà-Servera, Ernesto Muñoz-Mahamud

**Affiliations:** 1 Present Address: Departament de Cirurgia i Especialitats Medicoquirúrgiques, Facultat de Medicina i Ciències de la Salut, Universitat de Barcelona (UB), Barcelona, Spain, Barcelona, Spain; 2 Department Orthopedic Surgery and Trauma, Hospital Clinic of Barcelona, University of Barcelona, and Hospital Quirón Barcelona, Barcelona, Spain; 3 Institut d’Investigació Biomèdica August Pi i Sunyer (IDIBAPS), Barcelona, Spain., Spain

**Keywords:** Rheumatoid arthritis, Cervical spine instability

## Abstract

**Purpose:**

Rheumatoid arthritis (RA) is a systemic disorder that affects the cervical spine (CS). Synovial inflammation can disrupt spinal stability, leading to conditions such as atlantoaxial and/or subaxial subluxation, vertical settling, and combined instability. Although symptoms may appear in a minority of patients, they are commonly observed in those with advanced diseases. Myelopathy can develop in about 2.5% of patients with long-standing RA. Surgical treatment is indicated for the presence of myelopathy, progressive neurological deficit and/or chronic untreatable pain. The objective of this study is to evaluate the long-term outcomes following surgical treatment of CS involvement in patients with RA and to review the existing literature.

**Materials and methods:**

The present study is a retrospective and descriptive review of 17 patients with cervical involvement caused by RA who underwent surgery between 2000 and 2022. Collected data comprised the type of cervical lesion, the surgical approach and the pre-surgical, post-surgical and current neurological status.

**Results:**

Most patients were women (70,58%) and the mean age at surgery was 51,17 years. Myelopathy was present in 12 patients at the time of surgery. Ten patients improved the post-surgical Ranawat score, while seven remained stable. One patient died from post-surgical complications (5,88% of fatal events), and four patients passed away during the follow-up period.

**Discussion and conclusions:**

Surgical treatment of the cervical manifestation of RA provides benefits, improving quality of life and/or detaining the progression of the neurological damage. Even though the results are encouraging, surgery is not risk-free.

## Introduction

Rheumatoid arthritis (RA) is an autoimmune and progressive disease that can affect various joints. It impacts about 1–2% of adults [1] more frequently in women with a proportion of 3:1 [2]. 86% of those affected will develop complications in the cervical spine (CS) at some point during their disease [2]more prevalently in the rheumatoid factor seropositive subgroup [3]. The average time from the diagnosis of RA to the onset of cervical involvement is 12.3 years [4]. 

The CS is the third most frequently affected site in RA. Chronic synovial inflammation leads to the proliferation of fibrovascular tissue and pannus formation, resulting in ligament and bone damage [2, 5]. Bone erosion accompanied by deterioration of the surrounding soft tissues leads to compromised spinal stability. This renders the CS more vulnerable to subluxation and changes in the alignment, predisposing to the development of considerable complications such as spinal cord compression, neurological deficits, cranial nerve impairment, and bulbar dysfunction [1, 2]. 

Most common alterations are involvement of the suboccipital or upper CS, which includes the occipital bone (C0), atlas (C1) and axis (C2); and of the subaxial or lower CS, consisting of the remaining cervical vertebrae (C3-C7) [1, 2]. 

Most patients remain asymptomatic during the disease, although some of them may present worsening of the basal state due to muscle atrophy, arthropathy and decreased range of motion of the CS, accompanied or not of neurological manifestations [2, 6]. 

Due to the higher morbidity and mortality in those affected, the optimal therapeutic approach remains controversial [2]. Early treatment with disease-modifying antirheumatic drugs (DMARDs) and biologic agents (BAs) appears to have reduced the incidence of CS involvement. However, it does not rule out the risk of disease progression in patients with pre-existing cervical damage [5, 7]. 

Surgery is indicated in presence of myelopathy, progressive neurological deficit or instability with risk of nerve or brainstem compression, and/or chronic untreatable pain resistant to analgesics. It may lead to an improvement in both symptoms and pain control [8]. 

The aim of this review is to evaluate the long-term results obtained after surgical treatment of CS affection due to RA in a series of patients with or without myelopathy. Additionally, a literature review is conducted to provide further insights into the subject.

## Materials and methods

We retrospectively analyzed the clinical database of patients treated by surgery between 2000 and 2022. The Hospital Research Ethics Committee (CEIm) approved the study, which was conducted in adherence to the tenets of the Declaration of Helsinki (HCB/2022/1284).

Patients operated, with any kind of surgical treatment, due to upper and/or lower CS deformities due to RA were included. Exclusion criteria were rheumatologic diseases other than RA such as diffuse idiopathic spine hiperostosis (DISH).

Collected data included age, sex, diagnosis, presence or not of myelopathy, type of surgery, operated levels, operation complications and follow-up, which was calculated as the difference in years between the date of operation and last consultation. The different CS deformities were classified into three groups (Table 1): atlantoaxial subluxation (AAS), vertical settling (VS) and subaxial subluxation (SAS). Combined instability (global) was considered as the sum of upper and lower cervical spine involvement.

Radiological findings with anteroposterior, lateral flexion and extension and/or open-mouth projections supplemented with CT and MRI studies were reviewed in preoperative, immediate postoperative and last consultations. To assess the degree of neurological impairment, Ranawat classification [9] (Table 2) was applied in the three clinical controls mentioned before.

**Table 1:** Definition and diagnosis of CS instabilities [1]

Type of instability	Definition	Radiologic diagnosis
AAS	Weaking or rupture of ligaments and subchondral bone erosion in the AAJ	AADI <3 mm PADI ≥14 mm
VS	Projection of the odontoid process above the foramen magnum	Chamberlain line > 3 mm and/or McGregor line > 4,5 mm
SAS	Subluxation in the joints C3-C7 due to the destruction of the joint surface and the ligaments between the processes spinosis	Horizontal displacement > 3,5 mm

AAS = atlantoaxial subluxation, SAS = subaxial subluxation, VS = vertical settling, AAJ = atlantoaxial joint; AADI = anterior atlanto-dental interval, PADI = posterior atlanto-dental interval

**Table 2:** Ranawat classification [9]

Class	Description
I	Pain, no neurologic deficit.
II	Subjective weakness, hyperreflexia, dysesthesias.
IIIa	Objective weakness, long-tract signs, ambulatory
IIIb	Objective weakness, long-tract signs, non-ambulatory

## Results

We included 17 patients who underwent surgery for deformities in their CS, of whom twelve were women (70,58%) and five men (29,41%). The mean age of the patients at surgery was 51,17 years (range: 29–85 years).

Five patients were affected by AAS; VS and SAS were present in two and three patients, respectively. Combined involvement of both upper and lower spine (global) was diagnosed in seven patients. Twelve patients presented myelopathy at the time of surgery (70,58%). Overall, four patients passed away during the follow-up due to non-surgical related causes such as advanced age and the natural process of the disease. One patient died due to post-surgical complications (5,88% of fatal events). Reviewed patient’s characteristics are found in Table 3.

**Table 3:**
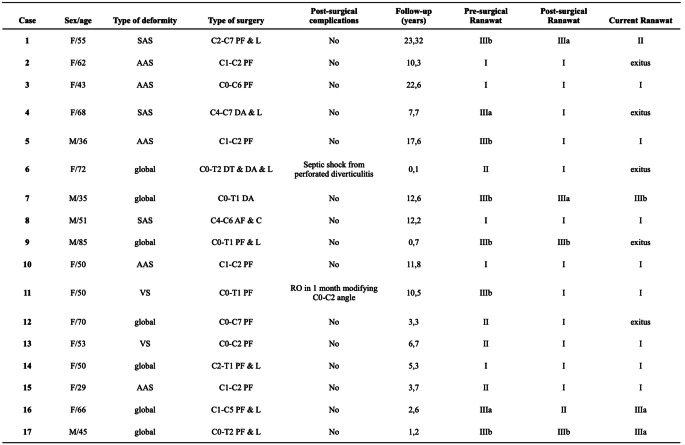
Reviewed patient’s characteristics. F = female, M = male, AAS = atlantoaxial subluxation, VS = vertical settling, SAS = subaxial subluxation, PF = posterior fusion, AF = anterior fusion, DA = double-approach fusion, DT = double-time, RO = re-operation, L = laminectomy, C = corporectomy

Patients underwent different surgical approaches: anterior fusion (one patient), posterior fusion (thirteen patients) and double-approach fusion (three patients). Laminectomy, to decompress the medullar canal, was performed in seven patients. Autologous bone graft, combined or not with allograft, was used to allow fusion in all patients.

Two patients were intervened in two different surgical times (cases 6 and 11). Case 6 had a previously planned double-time operation and case 11 had to be reoperated without prior planning to modify the cranio-cervical angle of an occipitocervical fixation. Post-surgical complications occurred in two patients, including an acute dysphagia that was resolved after three weeks (case 11) and a perforated diverticulitis (case 6), which was the only fatal one.

**Fig. 1:**
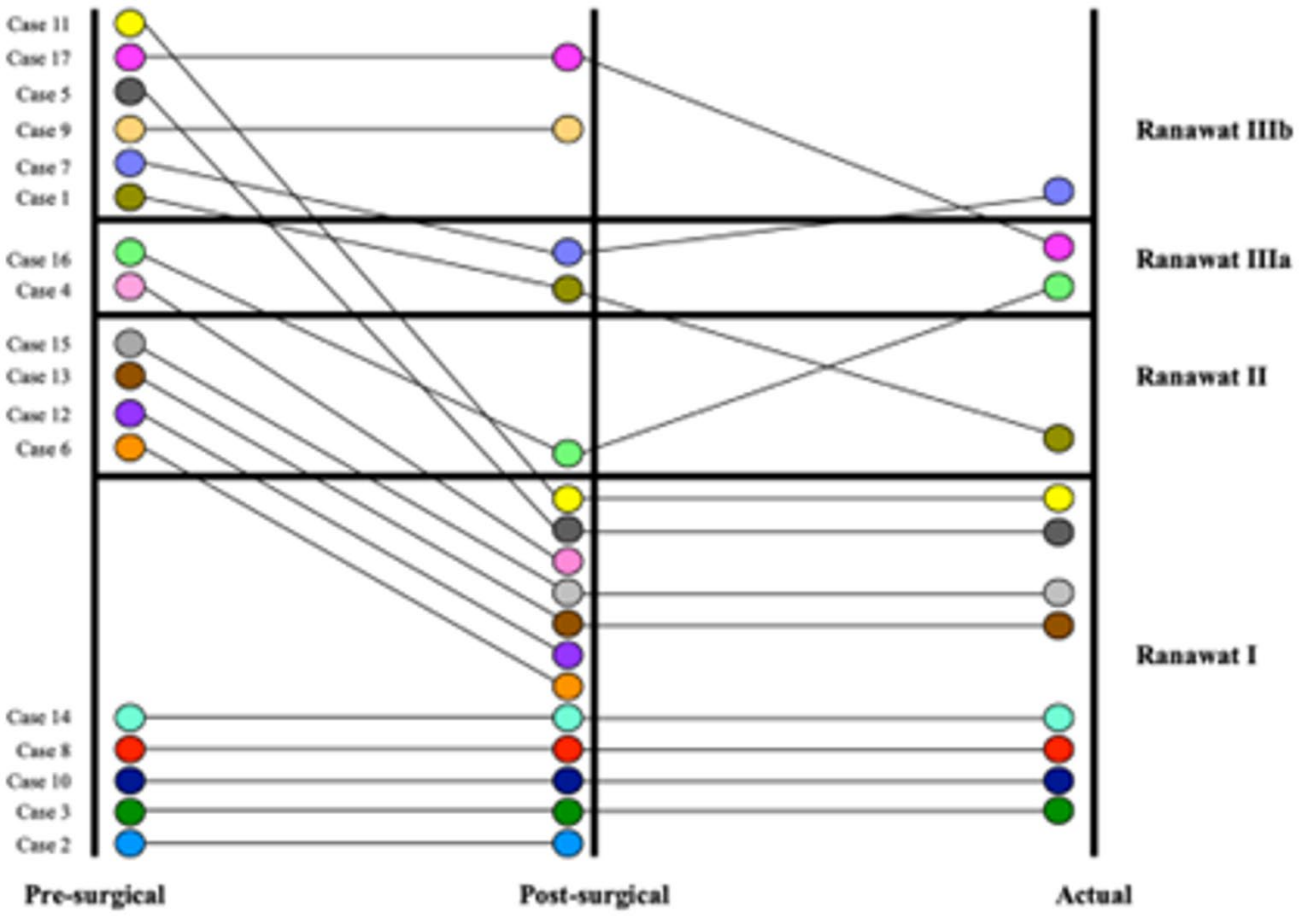
Preoperative, postoperative and current Ranawat status of patients in Table 3

All patients staged as Ranawat I before surgery showed no deterioration of their neurological status (5 patients). A preoperative Ranawat II was observed in four patients and all of them improved after surgery reaching a Ranawat I until the last visit, except for two of them who died (one of them due to post-surgical complications - case 6-). Two patients presented a pre-surgical Ranawat IIIA and all of them improved after the surgical treatment. Six patients (29,41%) were bedridden or required wheelchair (Ranawat IIIB) prior to surgery, of which four improved, although the rest of them (cases 9 and 17) remained the same. Preoperative, postoperative and actual Ranawat score results are shown in Fig. 1.

**Fig. 2:**
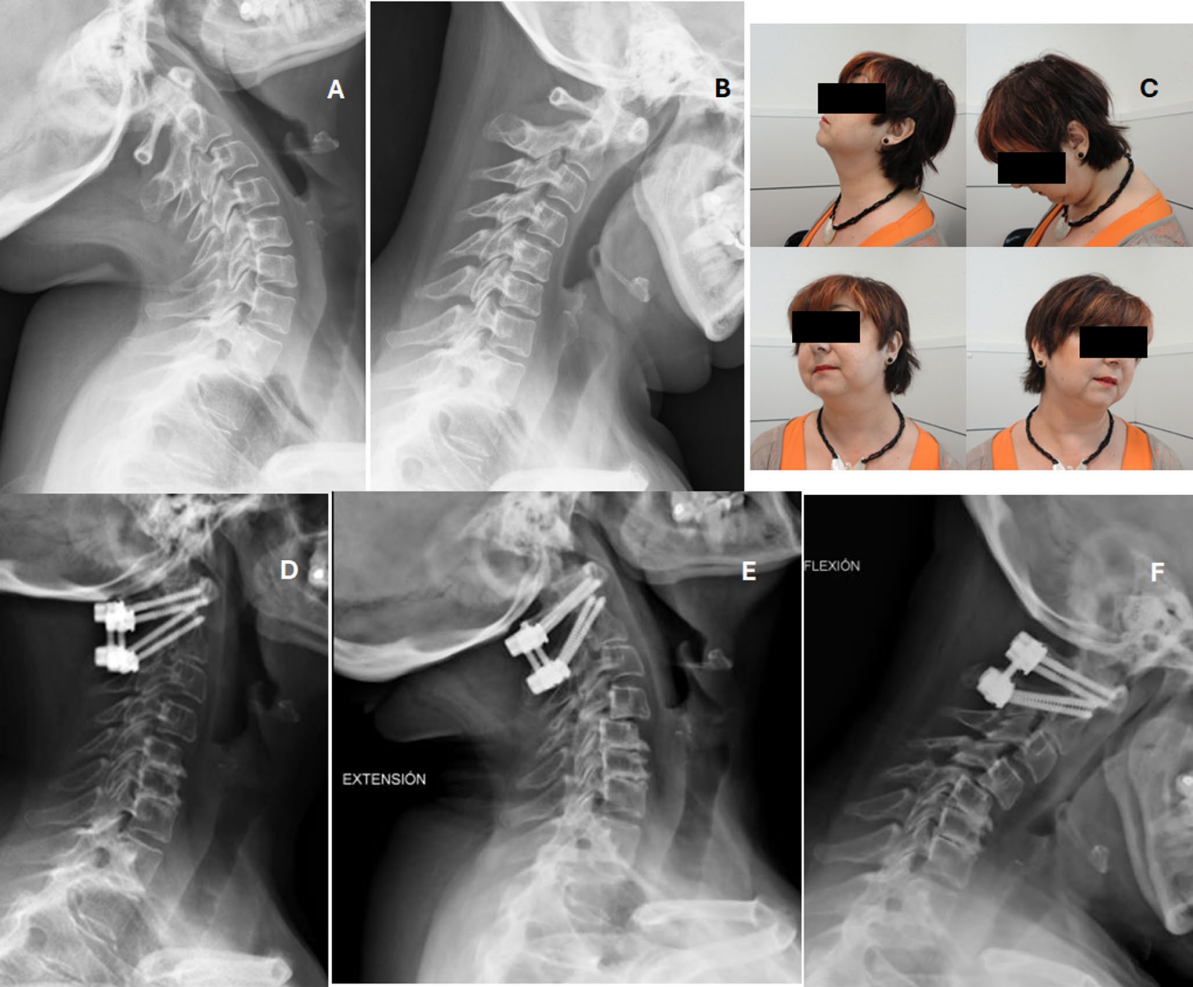
Case 10, Table 3. 50-year-old female with reducible Atlanto-Axial subluxation without neurological affectation. **A** and **B**: dynamic flexion-extension shows Atlanto-Axial instability. There was no subaxial involvement at the year 2010; **C**: Clinical results at six months post-operation; **D**: Five-year follow-up lateral XR shows C5-6-7 affectation and a slight subluxation C4-5 in this static radiograph; **E** and **F**: In January 2025, fifteen years after the index operation, an instability C4-C5 due to the progression of the disease is clearly seen in dynamic radiographs

**Fig. 3:**
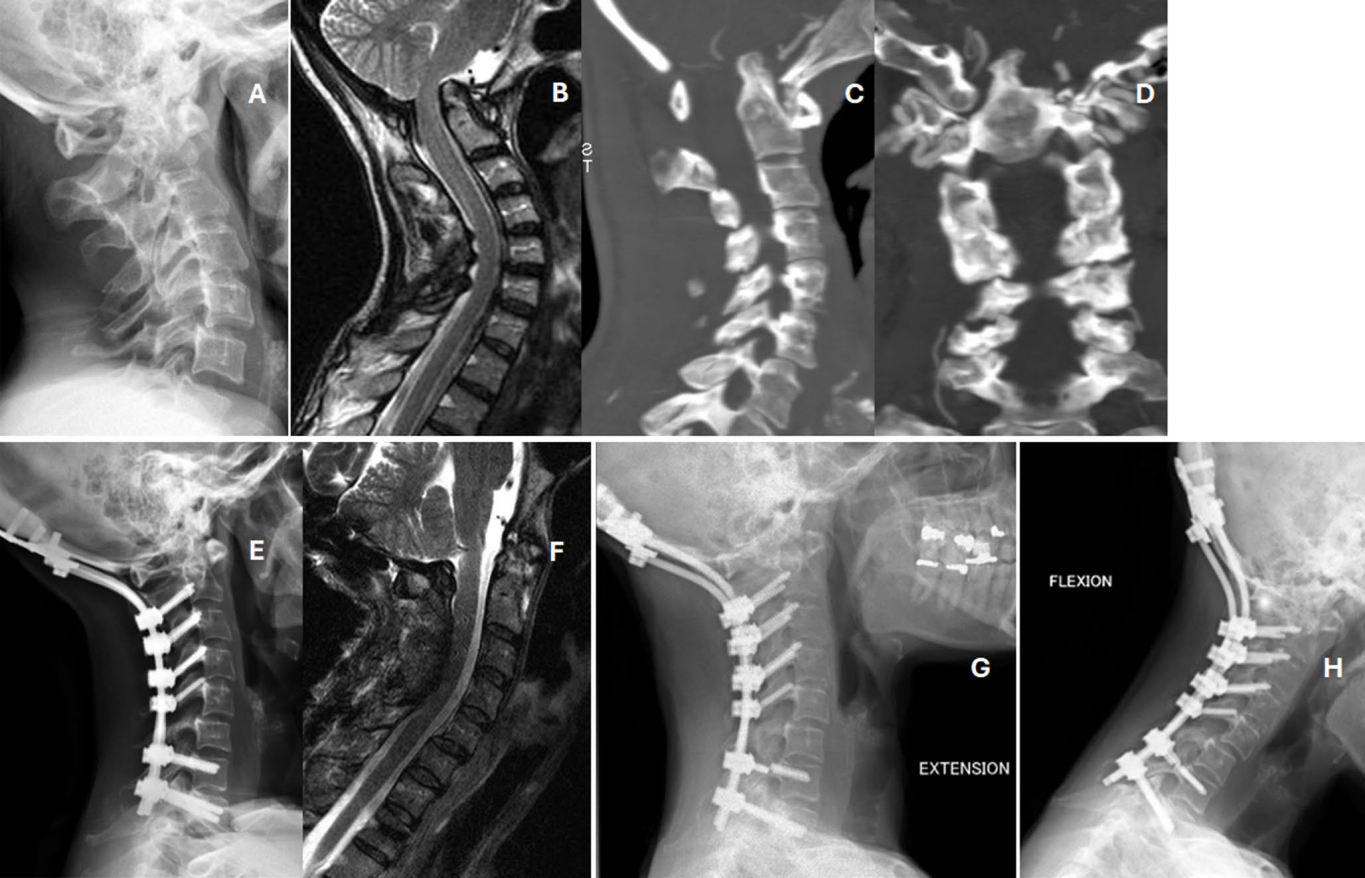
Case 11 Table 3. **A**: 50-year-old female with vertical settling, fixed flexion deformity and non-reducible atlanto-axial dislocation in flexion-extension radiographs. Also combines a C5-C6 subluxation. Neurological status Ranawat IIIB; **B**: MRI shows cranial displacement of the odontoid process with brainstem compression; **C** and **D**: Sagittal and coronal angio-CT scan. Odontoid process is prominent into the foramen magnum; **E** and **F** Postoperative XR and MRI showing correction of the deformity and indirect neurological decompression due to deformity correction—no laminectomy- (2011); **G** and **H**: Flexion-extension radiographs in November 2024, broken screws at C7 five years ago

Six patients are currently stable (cases 4, 6, 8, 11, 17 and 18) and two are progressing to subaxial involvement (cases 10 and 13) (Fig. 2). One patient has suffered a screw breakage eight years after surgery (case 11) without consequences (Fig. 3). Another patient has endured mobilization of the implanted surgical material years after the operation (case 16).

## Discussion

RA is a chronic and progressive autoimmune disease that affects the CS leading to significant deformities. CS impairment can occur in the suboccipital or upper spine as well as in the subaxial or lower spine.

Degeneration of the upper spine joints typically presents increased sliding motion between C1-C2 vertebrae, thus triggering instability that may degenerate in late stages into subluxation [4]. Instability of the AAJ can be classified as reducible, partially reducible or fixed, according to flexion-extension radiographies and/or the response to traction. AAS is the most frequent type of subluxation of the AAJ (75%) [1, 10], which is the most frequently affected in early stages of the disease [4, 11]. AAS can be radiologically graded measuring the anterior atlanto-dental interval (AADI) and the posterior atlanto-dental interval (PADI) in flexion-extension radiographies and/or Computed Tomography (CT) or Magnetic Resonance Imaging (MRI). AADI consists in the horizontal distance from the posterior aspect of the anterior arch of C1 to the anterior cortex of the dens in midplane. PADI is the space available for the spinal cord, that if it is less than 14 mm it can cause compression of the medulla. Other projections may be useful in the AAS diagnosis or evaluation, for example, open-mouth radiography is used to assess the odontoid process [1, 10].

The projection of the odontoid process above the foramen magnum indicates VS, cranial settling or basilar impression, and it is a consequence of a C0, C1 and C2 joint erosion [1, 6]. VS occurs in 15–20% of patients with CS involvement due to RA, and it elevates the risk of brain compression and sudden death [2, 12] (Fig. 3). The diagnosis of VS can be established by different diagnostic criteria such as Chamberlain, McGregor and Redlund-Johnell. Chamberlain’s line connects the posterior border of the hard palate to the posterior portion of the foramen magnum. The McGregor line links the hard palate to the most caudal point of the occipital curve. Finally, the Redlund-Johnell criteria is defined as the distance between the centre of the lower cover plate C2 and the McGregor line [1]. 

As for the involvement of the subaxial spine, it could relate to the affection of the upper spine. The rheumatoid lower spine can be presented as single or multiple SAS with or without associated kyphosis. Facet joints of the subaxial spine are also truly synovial and may be affected by the disease [4]. A 3.5 mm displacement of subaxial vertebrae on a lateral functional projection radiograph is considered diagnostic of instability [10]. 

Most patients with either of these deformities may remain asymptomatic. However, patients diagnosed with AAS may report a sensation of forward head droop when the head is flexed. Additionally, there may be crepitus and a “clunking” sound related to the sensation of cranial drooping. Occipital nerve compression secondary to AAS or VS can contribute to the development of occipital headaches. Migraines and neck pain may occur due to compression of the C2 nerve root or the greater auricular nerve. Rarely patients will develop swallowing and/or phonatory manifestations [2, 6]. 

Myelopathy occurs in patients with spinal cord compression. The main signs and symptoms of this condition are muscle weakness and atrophy, hyperreflexia, paraesthesia in the extremities, loss of proprioception and bladder dysfunction [2]. Recently has been reported that severe AAS can cause trauma to the tectorial membrane and duramater due to repetitive forced flexion head movements, developing consequently a subdural haemorrhage [13]. 

**Fig. 4:**
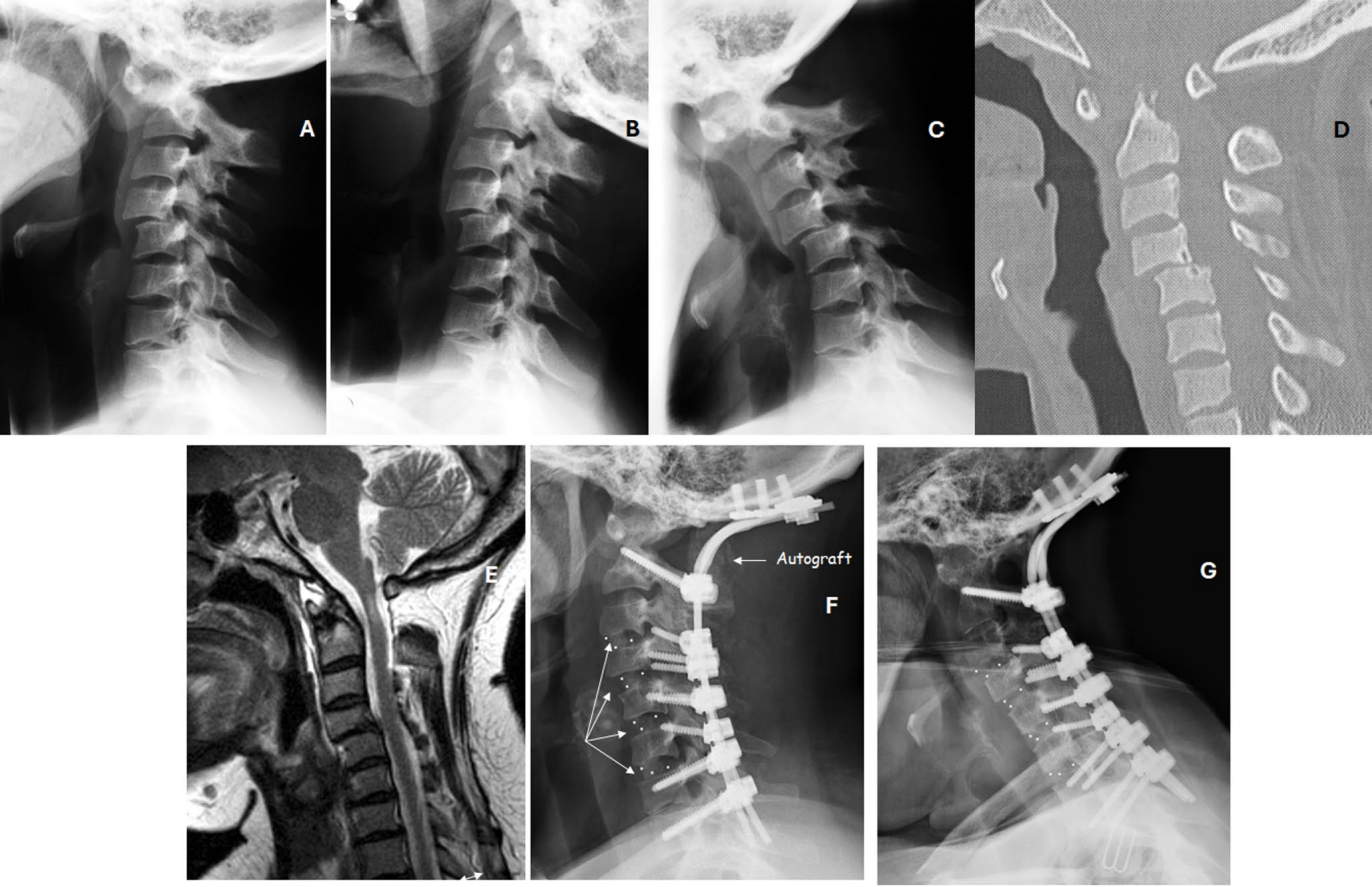
Case 7 Table 3. **A**-**B** and **C**: 35-year-old female with reducible Atlanto-Axial subluxation and non-reducible C4-C5 subluxation in flexion-extension radiographs, with neurological affectation Grade III in the Ranawat classification; **D**: CT scan; **E**: MRI in which severe myelopathy signal at C1-C2 level; **F**: Lateral radiography after a double approach, first posterior, occipito-T1 fixation with correction of the deformity (white arrows shows cages with autograft at C3-4-5-6-7 after discectomy); Autograft at Occipit-C2; **G**: Lateral XR eleven years after index operation showing slight kyphosis at cervicothoracic level

When a patient is presented with symptomatology and/or radiographs suggestive of cervical manifestation of RA, the affectation of soft tissues and bone structures should be evaluated by CT and/or MRI. CT is usually well-worn to assess the bone structure of the CS, but it can be helpful to evaluate soft tissues inflammation when MRI is contraindicated. Atypical rotatory or lateral subluxation of the AAJ is best detected with CT, and it is also useful in surgical treatment planning [6, 10, 13]. Although CT is effective, MRI is the test of choice in the evaluation of the CS in patients with RA. Ellatif et al. [6] asserts MRI evidences the presence of an occult pannus of > 3 mm on radiographs in two-thirds of patients with AAS. The main benefit of MRI is the possibility of detecting spinal cord or nerve compression as well as synovitis, erosions and pannus [6] (Fig. 4).

In the 1970s, conservative treatment was considered the established standard of care for this condition. Over several decades, authors such as Isdale and Conlon et al. [14] and Smith et al. [15] shared the consensus that surgery was not justified unless there was evidence of vascular or neurological impairment. Other authors such as Ranawat et al. [9] and Ferlic et al. [16] reported high rates of post-surgical fatal events. Contrary to this opinion, in the 2000s, authors like Shen et al. [17] put forth the view that surgical treatment should be carefully evaluated for each individual case. The same author also concluded that initiating prophylactic fusion in asymptomatic patients with radiological evidence of CS involvement could decrease the risk of paralysis, albeit with increased morbidity and mortality added by the intervention. Nowadays, surgical treatment of these deformities seems to be performed when there is myelopathy with progressive neurological affection or when analgesic treatment is not enough for pain control [8]. However, Schmitt-Sody et al. [28] advocate prophylactic surgical intervention in their conclusion. In 2021, Shlobin and Dahdaleh et al. [2] affirmed that surgical management of AAS and SAS was superior to conservative management, providing greater benefits particularly in the AAS group.

Early and appropriate treatment is crucial in preventing the progression of rheumatoid cervical disease [18]. With the use of DMARDs and BAs, the prevalence of CS lesions is decreasing, although the incidence remains high (> 30%) [1, 18]. However, despite receiving this treatment, patients with RA still have a higher risk of developing neurological complications and sudden death [19]. In cases of advanced myelopathy, pharmacotherapy is ineffective in slowing down the progression of cervical structural damage, irrespective of the systemic activity of RA [18].

In the current patient series, surgery had a significant impact, with the majority of patients (95,12%) experiencing improvement or maintaining stability in their quality-of-life post-operation. Among patients, only five were male, and three of them presented advanced neurological impairment (Ranawat IIIB). Male sex, among other factors, has been identified as an independent risk factor for the progression of cervical involvement [20]. 

In the AAS group, posterior fusion without laminectomy was performed in five cases, without any post-surgical complications. Currently, four patients are still alive, while one (case 2) passed away 10,3 years after surgery. Furthermore, one patient (case 10) is experiencing progression to SAS (Fig. 2). Several authors have described the degeneration of the subaxial spine following surgical treatment of the upper spine. Matsunanga et al. concluded that SAS occurrence after occipitocervical fusion associated with C1 laminectomy was significantly higher in operated patients than in the non-intervened ones [21]. It has also been described that operations on the C0-C2 and C1-C2 joints using pedicle screws may lead to SAS ten years later, requiring a re-operation [11]. However, these lesions may arise due to increased patient survival following surgical treatment and may represent the natural progression of the disease itself [21]. MacDowall et al. [12] recently concluded that prophylactic surgery of the subaxial spine is unnecessary following upper spine surgery.

AAS carries an eightfold higher mortality risk compared to patients with RA who do not have this condition [22]. Untreated myelopathy leads to loss of ambulation within three years from its onset, with a 0% survival rate within the first eight years [21]. The C1-C2 joint has the greatest motion in the CS, which becomes accentuated when pathological instability is present [23]. Surgery is indicated in AAS patients to reduce subluxation, stabilize the column, and prevent or halt neurological injury. It is also indicated to delay the degeneration to SAS. Atlantoaxial fusion via C1-C2 fixation is the means of choice in patients with isolated AAS [1]. Ideally, a reduction should be performed prior to fusion. In those cases where reduction is not possible, laminectomy may be helpful [23]. 

Kaito et al. [24] recently reported that pre-existing AAS is an independent risk factor for the development of VS. Aggressive intervention is crucial in VS patients due to the associated poor prognosis and higher mortality [17]. According to Ferrante et al. [25], spinal cord decompression can be obtained by directly resecting the odontoid process transorally or transnasally in symptomatic patients. However, facet spacers described by Goel et al. [26] and the use of polyaxial screws have replaced anterior transoral resection in the surgical treatment of VS [12].

SAS is a late manifestation of subaxial facet joint destabilization in RA, presenting as a “staircase deformity” with multilevel spondylolisthesis. This extended cervical involvement can explain the preference of long instrumentations with fixation extending from C0 to T2, rather than short ones, in cases of SAS associated to an AAS [12].

The surgical approaches for SAS cases varied widely. Case 1 underwent posterior fusion with laminectomy, case 4 had a double approach fusion combined with laminectomy and case 8 underwent an anterior fusion without a laminectomy. In patients with RA undergoing anterior fusion, the risks of post-surgical infection and the requirement to undergo revision surgery are 1.5 and 1.85 times higher, respectively, compared to the general population [27]. Two patients with SAS in this series of cases were affected of myelopathy, with a preoperative Ranawat IIIA (case 4) and IIIB (case 1). Both patients improved after surgery, even though one of them was exitus seven years after surgery due to operation non-related causes. Alive surgically treated patients with SAS remain stable nowadays.

Global involvement was present in seven patients in the current series of cases. Posterior fusion was performed in five patients and double-approach fusion in two patients. Five patients underwent laminectomy. Both post-surgical complications occurred in two patients with global involvement. These patients coincide in the type of involvement, double-time and double-track surgery. In patients with global affectation, surgical management must include a combination of both axial and subaxial spine surgery.

According to Janssen et al. [1], about 2.5% of patients with RA who have had the condition for more than 14 years will develop myelopathy. There is an increase of the perioperative morbidity and mortality of the non-ambulatory patients [1]. In a systematic review, Wolfs et al. [19] reported a poor neurological prognosis for patients with a preoperative Ranawat IIIB: only 38% of these patients recovered to ambulation. The results of a recent cohort of patients concluded a significant improvement in quality of life and pain after surgical treatment [12]. In this same cohort, it was found that myelopathy was mostly improved in patients with AAS [12]. In our series of cases, 70,58% of the patients presented with myelopathy at the time of surgery. This high incidence of neurological compromise can probably be explained due to the restricted indications of surgery. Patients without neurological compromise are not usually referred to orthopaedic surgeons.

Patients with RA have a significant risk of perioperative complications because of their cardiovascular and pulmonary compromise. This impacts considerably on their prognosis, so it should be considered in preoperative and postoperative management [5]. The regular medication (glucocorticoids, DMARDs and BAs) used by patients with RA can impact their surgical outcomes. These patients have higher frequency of surgical complications in comparison with the general population. The same applies to revision surgery [10, 27]. 

As with any surgical procedure, cervical fusion is not risk-free. Pseudarthrosis and osteopenia can lead to failure of the fusion resulting in subluxation of the joint around the fusion [6]. In the present case series, case 11 underwent an unplanned reoperation to modify the occipitocervical angle tone week later.

Post-operative halo collars and/or vests are recommended by some authors [6] in order to procure fusion in patients who have undergone surgery. This technique could be helpful in discarding possible pseudarthrosis that could compromise the union [6]. In the current series of cases it was not used in any case.

Mortality during follow-up in the abovementioned MacDowall et al. [12] study was 35% out of the 176 patients. In our case series, a total of four patients (23,52%) died during follow-up.

The limitations of the present study include a small sample size, retrospective design, and the lack of clinical data on disease activity. The inclusion of only surgically treated patients may be a potential methodology bias. Nevertheless, this study provides useful information on surgical treatment in rheumatoid cervical disorders.

## Conclusions

In conclusion, surgical treatment of the cervical manifestation of RA provides benefits, improving quality of life and/or stopping the progression of the neurological damage. The outcomes of this case series demonstrate an improvement in patients with already established and/or advanced myelopathy.

Even though the results are encouraging, surgery is not risk-free, increasing morbimortality either due to surgical stress, the surgical procedure or the disease itself.

## Data Availability

No datasets were generated or analysed during the current study.
